# Trans-Cinnamaldehyde Inhibits IL-1*β*-Stimulated Inflammation in Chondrocytes by Suppressing NF-*κ*B and p38-JNK Pathways and Exerts Chondrocyte Protective Effects in a Rat Model of Osteoarthritis

**DOI:** 10.1155/2019/4039472

**Published:** 2019-05-08

**Authors:** Tianwei Xia, Runzi Gao, Guowei Zhou, Jinzhu Liu, Jinsheng Li, Jirong Shen

**Affiliations:** ^1^The Affiliated Hospital of Nanjing University of Chinese Medicine, Nanjing 210023, China; ^2^School of Materials Science and Engineering, Southwest Jiaotong University, Chengdu 610031, China; ^3^Department of Traumatology and Orthopedics, Jiangsu Traditional Chinese Medicine Hospital, Nanjing 210029, China

## Abstract

**Objective:**

Trans-cinnamaldehyde (TCA), a compound from Cinnamomum cassia Presl, has been reported to have anti-inflammatory effect. However, its effect on cartilage degradation in osteoarthritis is unclear. This study is designed to examine the effects of TCA on cartilage in vitro and in vivo.

**Material and Methods:**

SW1353 cells and human primary chondrocytes were treated with varying concentrations of TCA (2-20 *μ*g/ml) for 2 h followed by IL-1*β* stimulation. Cell viability was examined by the MTT assay. Expression of MMP-1, MMP-3, MMP-13, ADAMTS-4, and ADAMTS-5 was examined by Western blot and RT-qPCR. Monosodium iodoacetate (MIA)-induced OA was established in rats to assess the chondrocyte protective effects of intraperitoneal injection of TCA (50 mg/kg).

**Results:**

TCA at a concentration of 10 *μ*g/ml had no significant effect on cell viability. MMP-1, MMP-3, MMP-13, ADAMTS-4, and ADAMTS-5 were decreased by TCA 2-10 *μ*g/ml in a dose-dependent manner (all* P*<0.05). Pretreatment with TCA decreased the degradation of I*κ*B*α* and increased the expression of p-I*κ*B*α*, indicating that NF-*κ*B inactivation was induced by TCA in IL-1*β*-stimulated SW1353 cells. Pretreatment with TCA decreased the levels of p-p38 and p-JNK, while the levels of p-ERK were not significantly affected. TCA 10 *μ*g/ml significantly decreased expression levels of MMP-1, MMP-3, MMP-13, ADAMTS-4, and ADAMTS-5. In vivo results showed that TCA alleviated cartilage destruction and the OARSI scores.

**Conclusion:**

TCA possesses anti-inflammatory effect in vitro and exerts chondrocyte protective effects in vivo, in which NF-*κ*B and p38-JNK were involved.

## 1. Background

Osteoarthritis (OA) is a degenerative joint disease that causes degenerative changes in articular cartilage and subchondral bone. OA is characterized by loss of articular cartilage, bone remodeling, and muscle weakness muscle weakness around the joints, resulting in knee joint pain, swelling, deformity, and instability [1]. OA affects approximately 40% of males and 47% of females throughout their lives and is a main cause of lower limb dysfunction in the elderly, resulting in a significant economic and social burden [2]. Abrasions, genetics, obesity, and other factors are recognized to be associated with OA [1]. The first-line therapies are based on acetaminophen and nonsteroidal anti-inflammatory drugs (NSAIDs) [3]. Despite improving management strategies, disease-modifying drugs for OA have not been discovered yet [3, 4].

**Figure 1:**
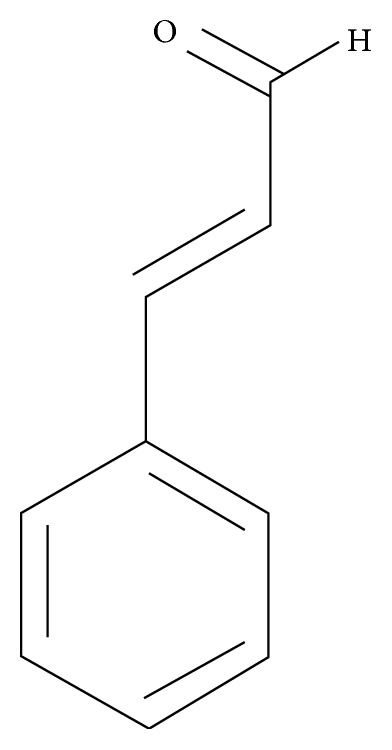
Chemical structure of trans-cinnamaldehyde (TCA).

Cinnamomum cassia Presl is derived from the traditional Chinese medicine and is used to treat inflammatory diseases, arrhythmia, ischemia, and so on [5–8]. The essential oil can alleviate inflammation in collagen-induced arthritis in BALB/c mouse models and in Freund's adjuvant-induced paw of ICR mice [9, 10]. TCA, the main bioactive component separated from the Cinnamomum cassia Presl essential oil, possesses potent anti-inflammatory activity in aging rats, dopaminergic degeneration of mice, endothelial cells, BV2 microglia, and monocytes/macrophages [11–15]. It also inhibits inflammatory damage in animal models of brain ischemia [16] and neuroinflammation [13]. Therefore, TCA is probably a potent anti-inflammatory compound with effects on the activation of cells involved in inflammatory diseases or injury. However, it is unclear whether TCA could be used to treat OA as an alternative medicine. Previous research has indicated that inflammatory cytokines like IL-1*β* play a pivotal part in OA pathogenesis [17], and IL-1*β* is mainly regulated by nuclear factor-kappa B (NF-*κ*B) [18]. IL-1*β* can also induce cartilage degradation by promoting the expression of a disintegrin and metalloproteinase with thrombospondin motifs (ADAMTS) and matrix metalloproteinases (MMPs) in chondrocytes [19–21]. Previous studies have indicated that members of the MAPK family, including p38 MAP kinases, c-Jun N-terminal kinase (JNK), and extracellular signal regulated kinase (ERK) play important roles in the cytokine regulation of MMP expression and subsequent cartilage destruction [22–24]. However, whether trans-cinnamaldehyde (TCA) (Figure 1) affects the expression of MMPs and ADAMTS via the NF-*κ*B and MAPK pathways has not been investigated yet.

In this study, we proposed the hypothesis that TCA could inhibit the inflammatory changes in chondrocytes and rat models of OA. The purpose of this research was to examine the effects of TCA on the chondrosarcoma-derived cell line and chondrocytes isolated from patients with OA and stimulated using IL-1*β*. In addition, we determined whether TCA had potential chondrocyte protective effects on monosodium iodoacetate (MIA)-induced OA in rats.

## 2. Materials and Methods

### 2.1. Cells and Primary Chondrocytes Isolation and Culture

The human chondrosarcoma-derived cell line SW1353 (SW1353 cells) (TCHu 128) was purchased from Cobioer Co., Ltd. (Shanghai, China). Human cartilage samples (tibial plateau and femoral condyle) were collected from eight patients with OA undergoing total knee replacement. Patients with OA were diagnosed on the basis of the American College of Rheumatology criteria. All samples came from the Department of Orthopedics, Jiangsu Traditional Chinese Medicine Hospital. Before the surgery, all of them got drug therapies, including NSAIDs (such as celecoxib, diclofenac, and ibuprofen). The age of subjects was 60-70. The duration of disease was from 5 to 10 years. The K-L scores were 3-4. This study was approved by the ethics committee of Jiangsu Traditional Chinese Medicine Hospital (October 26, 2017; approval number 2017NL-069-03). All procedures were in line with the ethical standards of the Human Experimental Committee (institutional and national) and the Helsinki Declaration of 1975, as amended in 2000. An informed consent form was signed by all patients before the trial.

To isolate the primary normal chondrocytes [25], the normal articular cartilage was cut into fine slices with a scalpel [26]. The pieces were treated overnight with type II Clostridium collagenase (lot# 1430519, Sangon Biotech, Shanghai, China) in DMEM/ F12 with 5% FBS (lot# 8118076, GIBCO, Invitrogen Inc., Carlsbad, CA, USA). The sample was filtered to remove the undigested cartilage and the chondrocytes were pelleted at 1500 rpm for 5 min before being resuspended [26]. After the initial isolation, the cells were maintained in high-density cultures in DMEM/F12 supplemented with 10% FBS, L-glutamine, 100 U/ml penicillin, and 100 *μ*g/ml streptomycin (GIBCO, Invitrogen Inc., Carlsbad, CA, USA) [26]. First-passage chondrocytes were obtained after 2 weeks. All experiments were done within 3 days of the passage 1 culture.

### 2.2. Cell Viability

The 3-(4, 5-dimethylthiazol-2-yl)-2, 5-diphenyltetrazolium bromide (MTT) assay was used to determine cell viability [27]. The cells were cultured at 5 × 10^3^ cells/well in 96-well plates. Cell cultures were treated with TCA (2, 5, 10, and 20 *μ*g/ml; purity >97%, lot# 18052735; Push Bio-Technology Co., Ltd., Chengdu, China) for 24 h. MTT was dissolved in PBS (0.5 mg/mL) and added to each well, and the cells were incubated at 37°C for 4 h. After removing culture media, DMSO was added to dissolve the formazan dye [28]. The optical density was measured at 490 nm.

### 2.3. RNA Extraction and Quantitative Real-Time PCR

Total RNA was isolated and purified from SW1353 cells and chondrocytes using a liquid nitrogen-chilled mortar and pestle, followed by TRIzol extraction [29]. Real-time PCR was performed by using the PrimeScript RT Reagent Kit (Takara Bio, Otsu, Japan), according to the manufacturer's instructions. Q-PCR was performed in an ABI Step One Plus instrument (Applied Biosystems, Foster City, CA, USA) using the SYBR Green PCR Master Mix (Thermo Fisher Scientific, Waltham, MA, USA). Gene expression levels were quantified according to the 2^−ΔΔCt^ method. All reactions were performed in triplicate and data were normalized to the human *β*-actin (Actb) gene [29]. The human primers were purchased from Dharmacon Inc. (Lafayette, CO, USA) (Table 1).

### 2.4. Whole Cell Protein Extraction and Western Blotting

Cells were lysed with the RIPA lysis buffer (50 mM Tris-HCl, pH 7.4, 150 mM NaCl, 0.5% EDTA, 0.5% sodium orthovanadate, 1% Triton X-100, and 0.1% SDS) containing protease and phosphatase inhibitors, incubated on ice for 20 min, and cleared by centrifugation (13,000 rpm for 5 min at 4°C) [29]. The protein concentration was measured with a bicinchoninic acid (BCA) protein assay kit (Pierce Chemical, Dallas, TX, USA) using bovine serum albumin (BSA) as the standard.

Subsequently, normalized volumes of samples (20 *μ*g of proteins) were isolated on 10% SDS-PAGE and transferred onto PVDF membranes (Bio-Rad, Richmond, CA), which were closed with 5% (w/v) milk for 1 h and probed with diluted antibodies overnight. Major antibodies were used as follows: anti-I*κ*B*α* (1:1000), anti-Phospho-I*κ*B*α* (1:1000), anti-GAPDH (1:1 000), anti-ERK (1:1000), anti- p38 (1:1000), anti-JNK (1:1000), anti-Phospho-ERK (1:1000), anti-Phospho-p38 (1:1000), and anti-Phospho-JNK(1:1000) (all from Santa Cruz Biotechnology, Santa Cruz, CA, USA) [30]. Proteins were detected using infrared dye-coupled secondary antibodies (1:4000) (goat anti-mouse IRdye800, goat anti-mouse IRdye680, goat anti-rabbit IRdye800, and goat anti-rabbit IRdye680) [31]. Detection was repeated for at least three times using WB analysis. The membranes were scanned using Tanon 5200 scanner (Tanon, Shanghai, China). Detection was repeated for at least three times using WB analysis. The intensities of protein bands were quantified by Image J software and the relative protein level was normalized to GAPDH [15].

### 2.5. Rat OA Model and Drug Administration

Twenty male Wistar rats (250-300 g) raised under pathogen free conditions were purchased from Beijing Vital River Laboratory Animal Technology Co., Ltd. The rats were randomized to four groups: MIA-induced group treated by TCA (ten rats, right knee), MIA-induced group treated by vehicle (ten rats, right knee), Sham MIA-induced group treated by TCA (ten rats, left knee), and MIA-induced group treated by vehicle (ten rats, right knee). OA-like arthritis was induced by intra-articular injection of MIA (1 mg/50 *μ*L normal saline per joint) into right knee joints. After anesthesia, a 29-gauge needle (Becton Dickson Co., Ltd., Franklin Lakes, NJ, USA) was used to inject the solution through the patellar ligament of the right knee. The Sham MIA injection group was induced by intra-articular injection of normal saline (NS) (50 *μ*L per joint) into left knee joints. From the next day, the rats were randomized to two groups: TCA group (ten rats) and control group (ten rats). The rats were injected intraperitoneally with TCA (purity >97%, lot# 18052735; Push Bio-Technology Co., Ltd., Chengdu, China) dissolved in oil (Arowna, Shanghai, China) (total of 300 *μ*L, 50 mg/kg of TCA solubilized in oil with 30 *μ*L of DMSO) or vehicle (total 300 *μ*L containing 270 *μ*L of oil and 30 *μ*L of DMSO), respectively, every day. All treatments lasted four weeks. The rats were sacrificed by cervical dislocation 24 h following the last injection.

### 2.6. Specimen Preparation, Histological Observation, and Evaluation

4% neutral buffered formalin was used to fix whole dissected knee joints for 48 h. Then they were washed with water, decalcified with 10% EDTA for 4 weeks, and embedded in paraffin. Sagittal sections (10-*μ*m thick) were obtained and colored by Safranin O-fast green. Slides were observed under the microscope and evaluated in the light of the Osteoarthritis Research Society International (OARSI) scores [32, 33]. At least 5 slides in each sample were used to independently evaluate the grade and stage of the knee joint. All slides were blindly assessed by two independent investigators. The product of the grade and stage values for each slide was used to define OA score. The histopathology scores were averaged for each knee joint before statistical analyses. The Institutional Animal Care and Use Committee (IACUC) of Jiangsu Traditional Chinese Medicine Hospital approved all procedures and protocols of vitro study. All methods and analyses were performed according to the relevant guidelines and regulation.

### 2.7. Statistical Analysis

The data were processed using SPSS 22.0 (IBM, Armonk, NY, USA). Continuous data were shown as means ± standard deviation and analyzed using one-way ANOVA with the Tukey's post hoc test or two-sample t-test for independent samples. Two-sided* P*-values <0.05 were considered statistically significant.

## 3. Results

### 3.1. High Concentration of TCA Decreases Cell Viability

**Figure 2:**
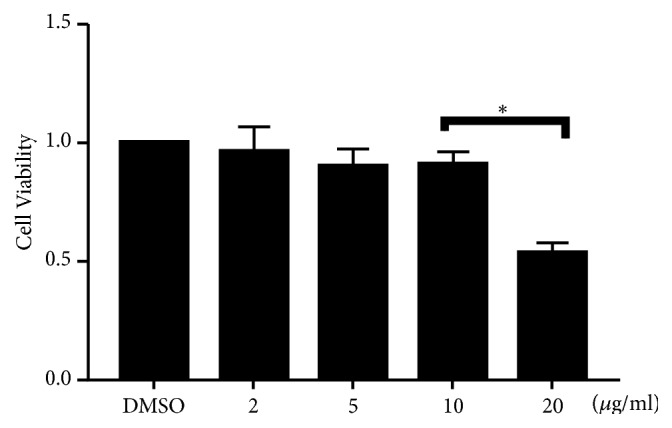
High concentration of TCA decreases cell viability. We initially examined the cytotoxicity of TCA to primary SW1353 cells and varying concentrations of TCA (2-20 *μ*g/ml) for 24 h. Each value indicates the mean ± SEM from three independent experiments. *∗P*<0.05.

We initially examined the cytotoxicity of TCA to primary SW1353 cells and varying concentrations of TCA (2-20 *μ*g/ml) for 24 h. The MTT assay showed that TCA at a concentration of 10 *μ*g/ml had no significant effect cell viability (Figure 2).

### 3.2. TCA Reduces the mRNA Expression of MMP-1, MMP-3, MMP-13, ADAMTS-4, and ADAMTS-5 in IL-1*β*-Stimulated SW1353 Cells

**Figure 3:**
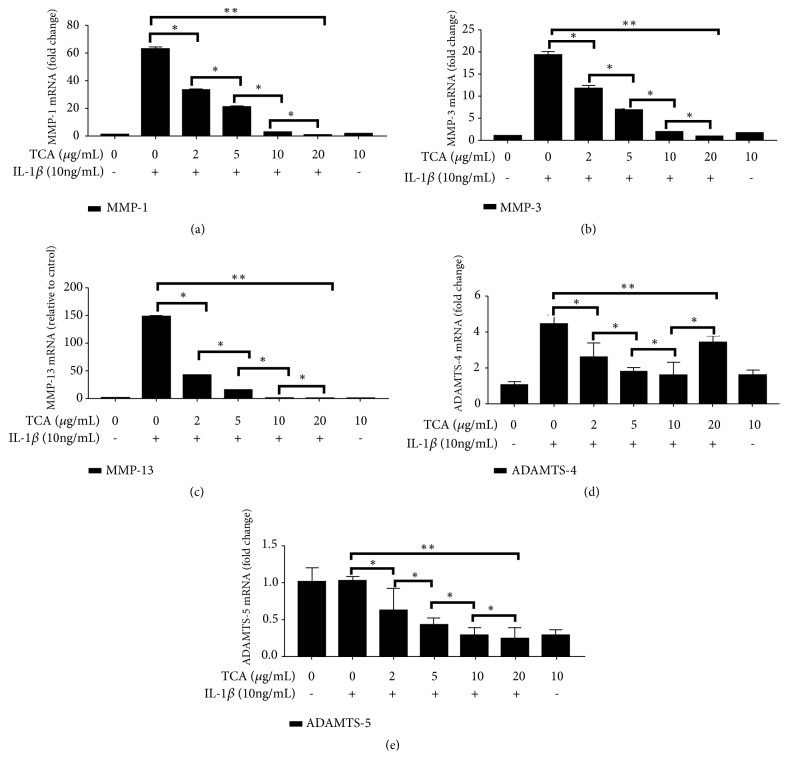
TCA reduces the mRNA expression of MMP-1, MMP-3, MMP-13, ADAMTS-4, and ADAMTS-5 in IL-1*β*-stimulated SW1353 cells. The SW1353 cells were pretreated with varying concentrations of TCA (2-20 *μ*g/ml) for 2 h followed by IL-1*β* (10ng/ml) stimulation for 6 h. The mRNA was assessed by RT-qPCR. (a) MMP-1. (b) MMP-3. (c) MMP-13. (d) ADAMTS-4. (e) ADAMTS-5. *∗P*<0.05; *∗∗P*<0.01.

The SW1353 cells were pretreated with TCA (10 *μ*g/ml) for 2 h followed by IL-1*β* (10 ng/ml) stimulation for 6 h. The expression levels of MMP-1, MMP-3, MMP-13, ADAMTS-4, and ADAMTS-5 were assessed by RT-qPCR assays. MMP-1, MMP-3, MMP-13, ADAMTS-4, and ADAMTS-5 were decreased by TCA 2-10 *μ*g/ml in a dose-dependent manner (all* P*<0.05). At 20 *μ*g/ml TCA, MMP-1, MMP-3, and MMP-13 expression levels were significantly lower than 10 *μ*g/ml TCA. ADAMTS-5, which is involved in cartilaginous matrix degradation, was also decreased when TCA was increased to 20 *μ*g/ml. However, ADAMTS-4 expression levels showed a significant increase with 20 *μ*g/ml TCA compared with 10 *μ*g/ml TCA (Figure 3).

### 3.3. TCA Suppresses NF-*κ*B Activation and I*κ*B Degradation in IL-1*β*-Stimulated SW1353 Cells

**Figure 4:**
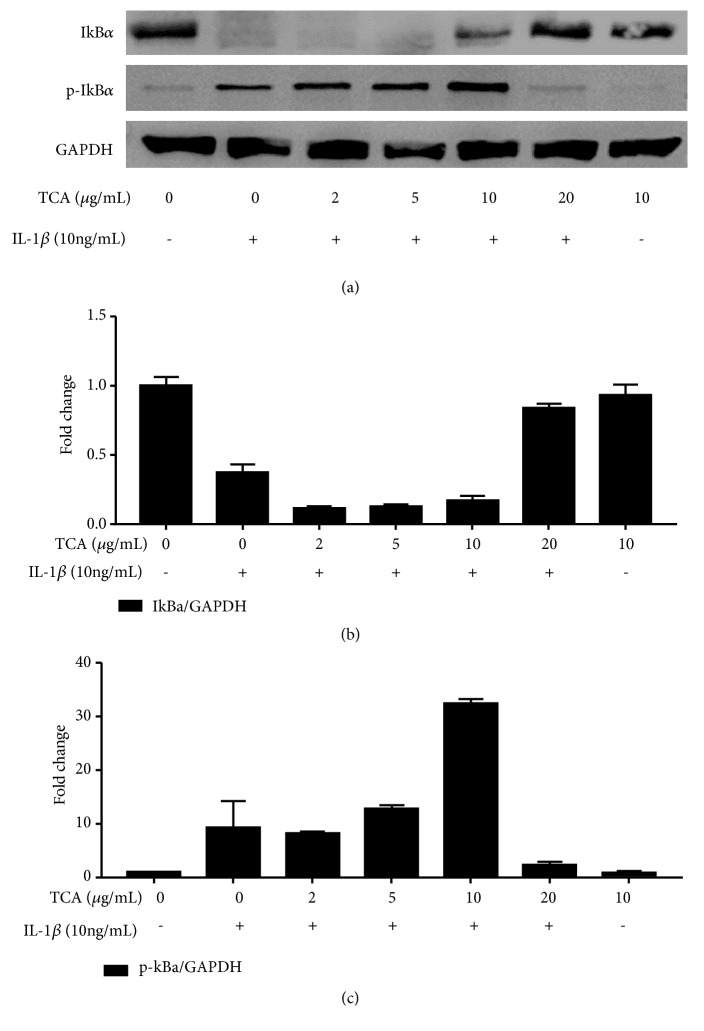
TCA suppresses NF-*κ*B activation and I*κ*B degradation in IL-1*β*-stimulated SW1353 cells. The SW1353 cells were preprocessed with varying concentrations of TCA (2-20 *μ*g/ml) for 2 h, then stimulated with IL-1*β* (10 ng/ml) for 6 h. The proteins were detected by Western blot. The protein expression of I*κ*B*α* (b) and p-I*κ*B*α* (c) was standardized according to the respective level of GAPDH protein. Value was used to express relative changes compared with control, which was set to 1. *∗ P *<0.05.

The activation of NF-*κ*B by IL-1*β* stimulation can induce the expression of proinflammatory mediators, leading to inflammation [34]. We investigated whether NF-*κ*B pathway was activated by IL-1*β* or inhibited by TCA using Western blot. Pretreatment with TCA (2-10 *μ*g/ml) decreased the levels of I*κ*B*α*. Moreover, pretreatment with TCA (2-10 *μ*g/ml) increased the levels of p-I*κ*B*α* in a dose-dependent manner. These consequences indicated the subsequent NF-*κ*B inactivation was induced by TCA in IL-1*β*-stimulated SW1353 cells (Figure 4).

### 3.4. TCA Suppresses p38- JNK Activation in IL-1*β*-Stimulated SW1353 Cells

**Figure 5:**
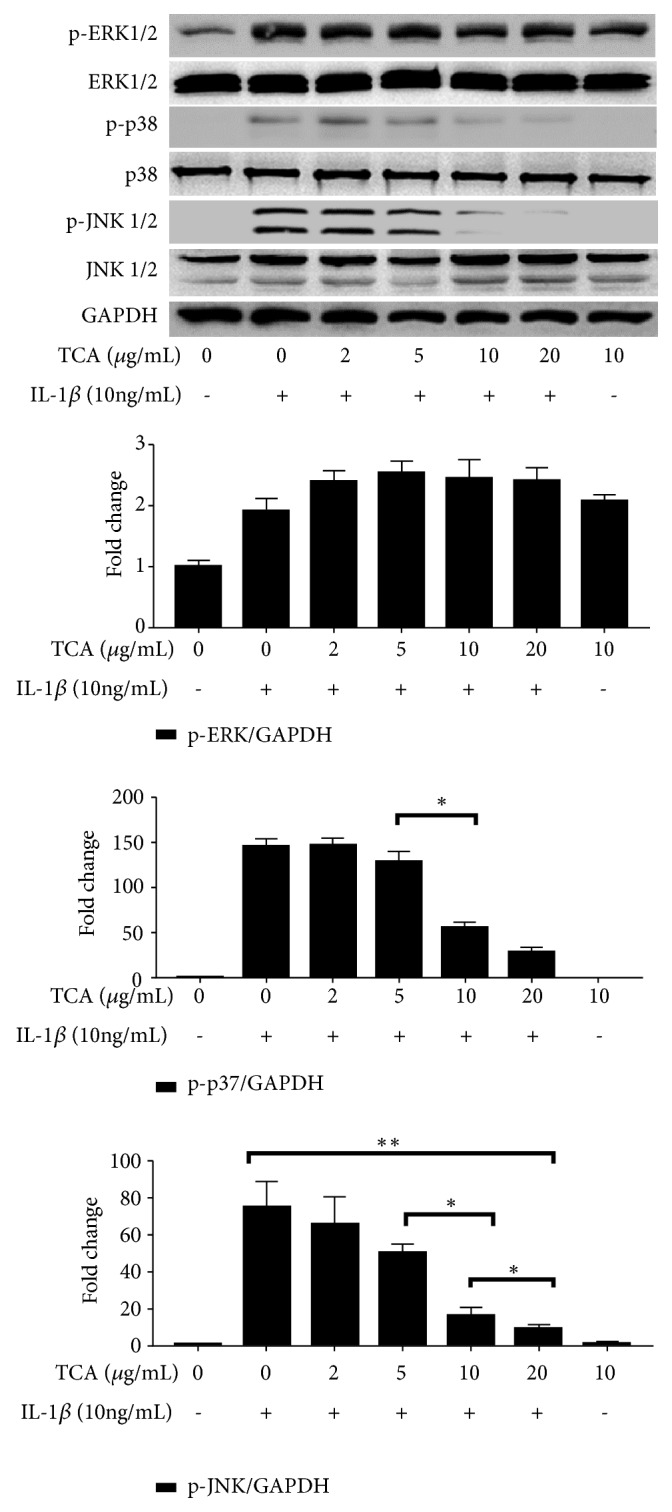
TCA suppresses p38-JNK* activation* in IL-1*β*-stimulated SW1353 cells. The SW1353 cells were preprocessed with varying concentrations of TCA (2-20 *μ*g/ml) for 2 h and then stimulated with IL-1*β* (10 ng/ml) for 6 h. The proteins were detected by Western blot. The protein expression of p-ERK (a), p-p38 (b), and p-JNK1/2 (c) was standardized based on the respective level of GAPDH protein. Value was expressed as relative changes in comparison to control, which was set to 1. *∗ P *< 0.05.

Next, we investigated the effects of TCA on the activation of the MAPK signaling pathways. Activated ERK, p38, and JNK1/2 were specifically assessed by Western blot. IL-1*β* induced an increase in the levels of p-ERK, p-p38, and p-JNK1/2, demonstrating that IL-1*β* was a potent activator of all three MAPK signaling pathways [15]. Intriguingly, TCA pretreatment reduced the levels of p-p38 and p-JNK1/2, while the expression of p-ERK had no significant effect (Figure 5).

### 3.5. TCA Reduces the mRNA Expression of MMP-1, MMP-3, MMP-13, ADAMTS-4, and ADAMTS-5 in IL-1*β*-Stimulated Chondrocytes

**Figure 6:**
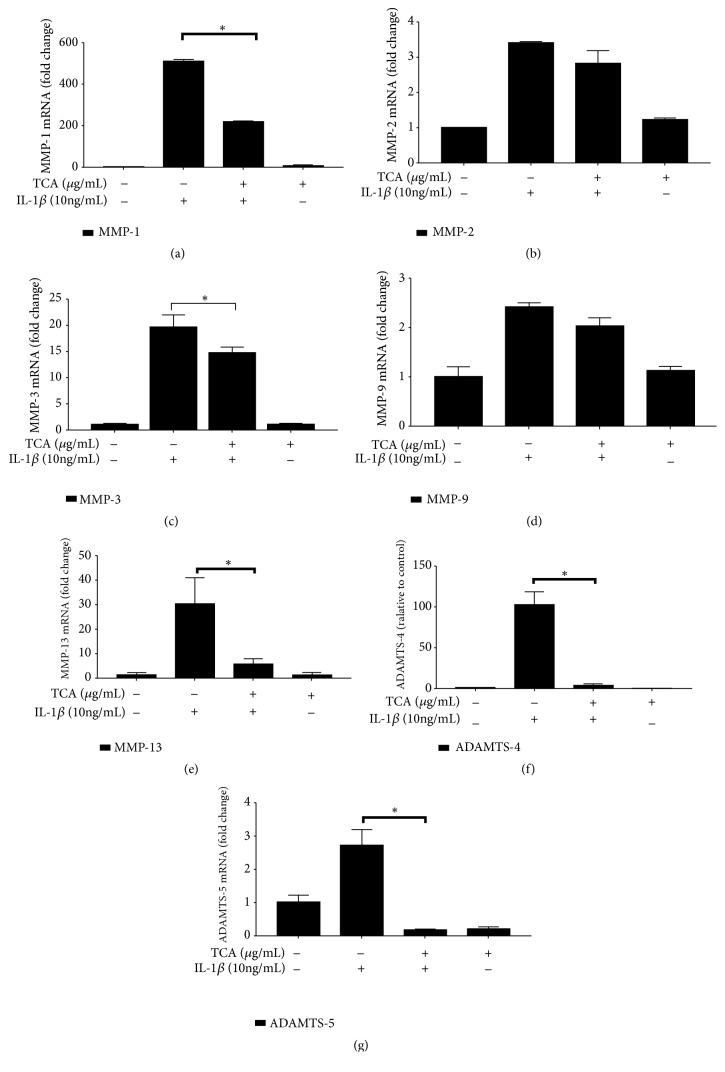
TCA reduced the mRNA expression of MMP-1, MMP-3, MMP-13, ADAMTS-4, and ADAMTS-5 in IL-1*β*-stimulated chondrocytes. The mRNAs were assessed by RT-qPCR. (a) MMP-1. (b) MMP-2. (c) MMP-3. (d) MMP-9. (e) MMP-13. (f) ADAMTS-4. (g) ADAMTS-5. *∗P*<0.05.

Since 10 *μ*g/ml TCA was the effective and safe concentration in SW1353 cells, we used this concentration to treat human primary chondrocytes for 2 h followed by IL-1*β* (10 ng/ml) stimulation for 6 h. The expression of MMP-1, MMP-2, MMP-3, MMP-9, MMP-13, ADAMTS-4, and ADAMTS-5 in chondrocytes was assessed by RT-qPCR. TCA 10 *μ*g/ml led to significant decreases in MMP-1, MMP-3, MMP-13, ADAMTS-4, and ADAMTS-5 expression levels compared with the vehicle group (Figure 6) (all* P*<0.05). However, MMP-2 and MMP-9 expression levels showed no obvious change compared with the vehicle group (Figure 6) (all* P*>0.05).

### 3.6. TCA Slows down the Progression of OA and Reduces the OARSI Scores In Vivo

**Figure 7:**
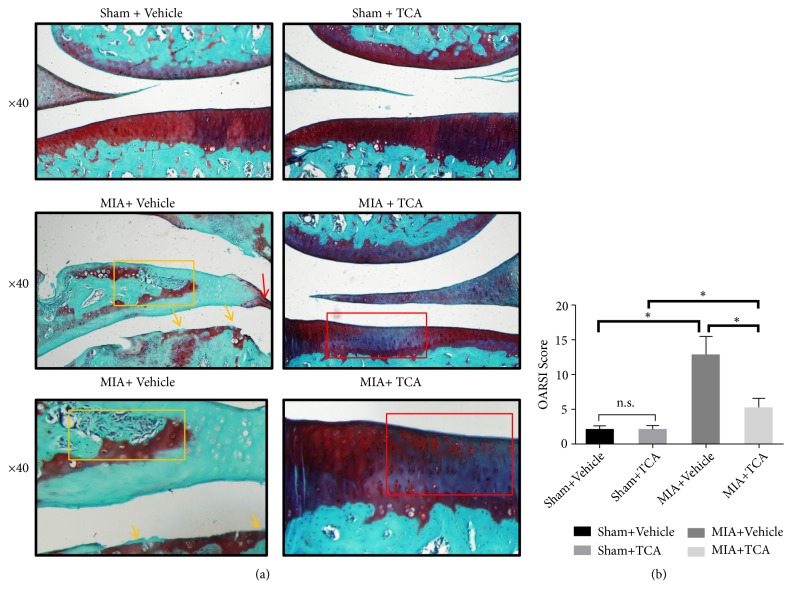
TCA exerts cartilage protective effects in MIA-induced rats after 4 weeks of intraperitoneal injection. (a) Knee joint sections were stained with Safranin O/fast green. The sections were from rats after 4 weeks administration of TCA or vehicle. Representative sections are shown. Formation of osteophytes (yellow arrow), severe disruption of meniscal tissue (yellow box), and hyperplasia of synoviocyte (red arrow) were observed in vehicle-treated MIA-induced rats. There was mild fibroid-like change in TCA-treated MIA-induced group (red box). (b) OARSI scores of articular cartilage at 4 weeks after surgery. *∗P*<0.05, NS: not statistically significant.

Safranin O/fast green staining showed that the articular surface of the two Sham MIA-injected groups was relatively smooth. The matrix and chondrocytes were divided into superficial, mid, and deep layers. Permanent matrix staining loss may be a result of cell death (necrosis and apoptosis). The OARSI scores of the two groups were low and there was no significant difference (*P*>0.05). The cartilage was near to denudation in the vehicle-treated MIA-induced group. The articular surface was mineralized cartilage or bone and loss of staining with Safranin O (Figure 7(a), lower left panel). Formation of osteophytes (yellow arrow), severe disruption of meniscal tissue (yellow box), and hyperplasia of synovium (red arrow) could also be observed (Figure 7(a), middle left panel; bottom left panel). OARSI scores were relatively high. In contrast, although there was mild fibroid-like change in TCA-treated MIA-induced group (red box), the superficial zone of the cartilage was smooth and Safranin O staining of matrix was abundant (Figure 7(a), middle right panel; bottom right panel). Compared with the vehicle-treated controls, OARSI scores in TCA-treated MIA-induced rats were significantly improved, and the difference was significant (*P*<0.05) (Figure 7(b)).

## 4. Discussion

It has been reported that TCA has anti-inflammatory effects [13, 16, 35], such as inhibiting the expression of inflammatory factors like IL-1*β*, NO, and PGE_2_ [13, 15, 35]. Nevertheless, whether TCA has benefits against cartilage degradation in OA-like inflammation is still unknown. Therefore, we examined the effects of TCA on OA-like conditions in vitro and in vivo. The results confirmed for the first time that TCA significantly suppressed IL-1*β*-induced mRNA expression of MMP-1, MMP-3, MMP-13, ADAMTS-4, and ADAMTS-5 in SW1353 cells and in human primary chondrocytes via suppressing NF-*κ*B and p38-JNK pathways.

An imbalance between chondrocyte anabolism and catabolism is the underlying mechanism of OA pathogenesis [1] and those mechanisms are highly correlated with inflammation [1]. It involves a variety of enzymes and cytokines [36]. IL-1*β* is an important cytokine proposed as a driver of OA and it induces catabolic gene expression in chondrocytes in vitro [37–39] and activates the NF-*κ*B, JNK, p38, and MAPK signaling pathways, leading to inflammation [34, 40–45]. Thus, chondrocytes stimulated with IL-1*β* in vitro are used to simulate the microenvironment in which OA occurs [45]. The results of the present study also proved the impact of IL-1*β* on NF-*κ*B and MAPK signaling pathway activation. It is worth noting that many studies reported the anti-inflammatory effects of TCA. Youn et al. indicated that TCA inhibits the expression of TLR2, TLR4, and MyD88 mRNA and downregulates the expression of COX-2 and IFN-*β* by inhibiting the oligomerization of TLR4 in the inflammation state [34]. In addition, TCA can play a role in strongly regulating monocyte/macrophage-mediated immune responses via thiolation of target cysteine residues in PI3K or PDK, which can also inhibit inflammation at the same time [46]. Kim observed that short-term (i.e., 10 days) feeding of CNA has a regulatory effect on age-related NF-*κ*B activation in aged rats. As the results showed, age-related activation of NF-*κ*B upregulated NF-*κ*B targeting genes, including inflammatory iNOS and COX-2, which were obviously downregulated in TCA-fed rats. Furthermore, he found that age-related NF-*κ*B activation was modulated by TCA via the NIK/IKK, ERK, and P38 MAPK pathways, all of which highlighted that TCA was an efficient antioxidant and may have broad applications prospects for proinflammatory NF-*κ*B and its dependent genes regulated by NF-*κ*B signaling [11]. However, he chose to exam kidney instead of cartilage because he believed it is vulnerable to oxidative stress and inflammation stimuli. Recently, the anti-inflammatory effects of TCA in LPS-induced macrophages produced via the suppression of mitogen-activated protein kinase (MAPKs) phosphorylation and proinflammatory genes were also revealed [35].

Previous study showed that IL-1*β* mediated induction of MMPs in chondrocytes [41, 47]. MMP family members are participated in the decomposition of the extracellular matrix (including proteoglycan and collagens) in normal physiological processes, but excess MMP activity leads to excess extracellular matrix destruction in OA [48]. Previous studies have demonstrated that MMP-1 and MMP-13 (especially MMP-13) are participated in the degradation of collagen II, which is a main component of the cartilage extracellular matrix. MMP-3 can help MMP-1 and MMP-13 degrade collagen II [49]. It is well known that the chondrocyte protective effects mediated by NF-*κ*B activation are connected with the inhibition of the MMP genes [50, 51]. The modulation of NF-*κ*B signaling has been suggested to be responsible for inhibiting IL-1*β*-induced expression of the MMP gene family in chondrocytes [52–54]. In this study, we observed that TCA inhibited IL-1*β*-induced I*κ*B degradation and suppressed the activation of p38 and JNK1/2 in SW1353 cells, suggesting that TCA may prevent inflammation induced by IL-1*β* in human OA chondrocytes by inhibiting those pathways. Overall, these results strongly suggest that TCA may possess anti-inflammatory and anticartilage degradation effects in cell models of OA. In this study, we also observed that TCA suppressed the mRNA expression of MMP-1, MMP-3, and MMP-13 in chondrocytes, which was induced by IL-1*β*. These data imply that TCA exerts chondrocyte protective effects on human OA chondrocytes by suppressing the production of MMPs. Aggrecan is also a main component of the cartilage extracellular matrix. Cleavage of aggrecan can be performed by several members of the ADAMTS family of metalloproteases [55]. ADAMTS-4 and ADAMTS-5 seem to be the most active aggrecanases [56]. The present study showed that TCA decreased the mRNA expression levels of ADAMTS-4 and ADAMTS-5, suggesting that TCA may slow down the progression of OA.

From another perspective, the results derived from an in vivo study demonstrate that TCA may possess cartilage protective effects in rat OA model. MIA-induced OA in rats induces rapid chondral erosion with progression of subchondral bone lesions that is similar to those of OA [57]. Both aggrecanase and MMPs play an important role in the cartilage degradation that occurs in this model [58]. This model could be used for studying chondrocyte protective drugs [59]. In view of the fact that TCA is barely soluble in normal saline and oil, we solubilized TCA in oil with the aid of DMSO (10% volume concentration). The histological observations revealed that TCA can be retained in the proteoglycans in cartilage and slow down the progression of OA in vivo. Considering that TCA is volatile and has poor water and oil solubility and low blood concentration, we believe that TCA cannot be made into a new drug for now. Considering that intraperitoneal injection is unsafe and easily susceptible for people, there would be a need to develop an orally administered form of TCA for the therapy of OA. Moreover, injections are more expensive and expensive than oral agents. Previous reports show that making TCA into a self-emulsifying nanoparticle emulsion or polylactic hydroxyacetic acid nanoparticles can improve its solubility, stability, and bioavailability [60, 61]. Structural modifications, inclusions, and modern formulation of TCA should be examined in the future. The toxicity of TCA also needs to be further studied.

## 5. Conclusion

Taken together, the results suggest that TCA at 10 *μ*g/ml decreases the expression of MMP-1, MMP-3, MMP-13, ADAMTS-4, and ADAMTS-5 in SW1353 cells and human chondrocytes isolated from patients with OA, without affecting cell viability. The results support that TCA can suppress NF-*κ*B and p38-JNK pathways and exert cartilage protective effects in cell models of OA. The results also support that TCA is able to significantly attenuate cartilage degradation in vivo. These results indicate that TCA may act as a promising anti-inflammatory agent with regard to OA.

## Figures and Tables

**Table 1:** Sequences of the primers used for RT-qPCR.

Primers	Forward primer (5′→3′)	Reverse primer (5′→3′)
MMP-1	ACTGAGAGAGGCTCCGAGAATG	GAACCCCGCATCTTGGCTT
MMP-2	TGATCTTGACCAGAATACCATCGA	GGCTTGCGAGGGAAGAAGTT
MMP-3	CTCTGGAGTAATGTCACACCTCT	TGTTGGTCCACCTTTCATCTTC
MMP-9	CCTGGAGACCTGAGAACCAATC	CCACCCGAGTGTAACCATAGC
MMP-13	AGTCTTCCAATCCTACTGTTGCT	TCCCCGTCACCTCCAATCC
ADAMTS-4	GAACATCGACCAACTCTACCCG	CAGGAGGAGATCGTGTTCCA
ADAMTS-5	GAGGAGGGAGATCGTGTTCCA	CAGCTCTAGTAGGCAGCGTC

## Data Availability

The data used to support the findings of this study are available from the corresponding author upon request.

## Abbreviations

TCA: Trans-cinnamaldehyde
MIA: Monosodium iodoacetate
OA: Osteoarthritis
NSAIDs: Nonsteroidal anti-inflammatory drugs
NF-*κ*B: Nuclear factor-kappa B
MMPs: Matrix metalloproteinases
ADAMTS: A disintegrin and metalloproteinase with thrombospondin motifs
MAPKs: Mitogen-activated protein kinase.

## References

[1] Gelber A. C. (2014). In the clinic. Osteoarthritis.. *Annals of Internal Medicine*.

[2] Johnson V. L., Hunter D. J. (2014). The epidemiology of osteoarthritis. *Best Practice & Research: Clinical Rheumatology*.

[3] Hochberg M. C., Altman R. D., April K. T. (2012). American College of Rheumatology 2012 recommendations for the use of nonpharmacologic and pharmacologic therapies in osteoarthritis of the hand, hip, and knee. *Arthritis Care & Research*.

[4] Berenbaum F., Eymard F., Houard X. (2013). Osteoarthritis, inflammation and obesity. *Current Opinion in Rheumatology*.

[5] Liao J.-C., Deng J.-S., Chiu C.-S. (2012). Anti-inflammatory activities of cinnamomum cassia constituents in vitro and in vivo. *Evidence-Based Complementary and Alternative Medicine*.

[6] Shimada Y., Goto H., Kogure T. (2000). Extract prepared from the bark of Cinnamomum cassia blume prevents glutamate-induced neuronal death in cultured cerebellar granule cells. *Phytotherapy Research*.

[7] Yu H.-S., Lee S.-Y., Jang C.-G. (2007). Involvement of 5-HT1A and GABAA receptors in the anxiolytic-like effects of Cinnamomum cassia in mice. *Pharmacology Biochemistry & Behavior*.

[8] Zaidi S. F., Aziz M., Muhammad J. S. (2015). Review: Diverse pharmacological properties of Cinnamomum cassia: A review. *Pakistan Journal of Pharmaceutical Sciences*.

[9] Sun L., Zong S., Li J. (2016). The essential oil from the twigs of Cinnamomum cassia Presl alleviates pain and inflammation in mice. *Journal of Ethnopharmacology*.

[10] Qadir M. M. F., Bhatti A., Ashraf M. U., Sandhu M. A., Anjum S., John P. (2018). Immunomodulatory and therapeutic role of Cinnamomum verum extracts in collagen-induced arthritic BALB/c mice. *Inflammopharmacology*.

[11] Kim D. H., Kim C. H., Kim M.-S. (2007). Suppression of age-related inflammatory NF-kappaB activation by cinnamaldehyde. *Biogerontology*.

[12] Wang F., Pu C., Zhou P. (2015). Cinnamaldehyde prevents endothelial dysfunction induced by high glucose by activating Nrf2. *Cellular Physiology and Biochemistry*.

[13] Fu Y., Yang P., Zhao Y. (2017). *trans*-Cinnamaldehyde Inhibits Microglial Activation and Improves Neuronal Survival against Neuroinflammation in BV2 Microglial Cells with Lipopolysaccharide Stimulation. *Evidence-Based Complementary and Alternative Medicine*.

[14] Chung J., Kim S., Lee H. A. (2018). Trans-cinnamic aldehyde inhibits Aggregatibacter actinomycetemcomitans-induced inflammation in THP-1–derived macrophages via autophagy activation. *Journal of Periodontology*.

[15] Zhang L., Zhang Z., Fu Y. (2016). Trans-cinnamaldehyde improves memory impairment by blocking microglial activation through the destabilization of iNOS mRNA in mice challenged with lipopolysaccharide. *Neuropharmacology*.

[16] Zhao J., Zhang X., Dong L. (2015). Cinnamaldehyde inhibits inflammation and brain damage in a mouse model of permanent cerebral ischaemia. *British Journal of Pharmacology*.

[17] Jotanovic Z., Mihelic R., Sestan B., Dembic Z. (2012). Role of interleukin-1 inhibitors in osteoarthritis: An evidence-based review. *Drugs & Aging*.

[18] Berenbaum F. (2004). Signaling transduction: Target in osteoarthritis. *Current Opinion in Rheumatology*.

[19] Yin W., Lei Y. (2018). Leonurine inhibits IL-1*β* induced inflammation in murine chondrocytes and ameliorates murine osteoarthritis. *International Immunopharmacology*.

[20] Zhang R., Wang C., Jiang H. (2019). Protective Effects of Sweroside on IL-1*β*-Induced Inflammation in Rat Articular Chondrocytes Through Suppression of NF-*κ*B and mTORC1 Signaling Pathway. *Inflammation*.

[21] Fernandes J. C., Martel-Pelletier J., Pelletier J. (2002). The role of cytokines in osteoarthritis pathophysiology. *Biorheology*.

[22] Thalhamer T., McGrath M. A., Harnett M. M. (2008). MAPKs and their relevance to arthritis and inflammation. *Rheumatology*.

[23] Wang P., Mao Z., Pan Q. (2018). Histone deacetylase-4 and histone deacetylase-8 regulate interleukin-1*β*-induced cartilage catabolic degradation through MAPK/JNK and ERK pathways. *International Journal of Molecular Medicine*.

[24] Jeong J.-W., Lee H. H., Choi E.-O. (2015). *Schisandrae Fructus* inhibits IL-1*β*-induced matrix metalloproteinases and inflammatory mediators production in SW1353 human chondrocytes by suppressing NF-*κ*B and MAPK activation. *Drug Development Research*.

[25] Gosset M., Berenbaum F., Thirion S., Jacques C. (2008). Primary culture and phenotyping of murine chondrocytes. *Nature Protocols*.

[26] Yang H., Wu D., Li H., Chen N., Shang Y. (2018). Downregulation of microRNA-448 inhibits IL-1*β*-induced cartilage degradation in human chondrocytes via upregulation of matrilin-3. *Cellular & Molecular Biology Letters*.

[27] Van Meerloo J., Kaspers G. J. L., Cloos J. (2011). Cell sensitivity assays: the MTT assay. *Methods in Molecular Biology*.

[28] Zheng L. T., Ock J., Kwon B.-M., Suk K. (2008). Suppressive effects of flavonoid fisetin on lipopolysaccharide-induced microglial activation and neurotoxicity. *International Immunopharmacology*.

[29] Zhou S., Lu W., Chen L. (2017). AMPK deficiency in chondrocytes accelerated the progression of instability-induced and ageing-associated osteoarthritis in adult mice. *Scientific Reports*.

[30] Kim B.-W., Koppula S., Kim I. S. (2011). Anti-neuroinflammatory activity of kamebakaurin from Isodon japonicus via inhibition of c-Jun NH 2-terminal kinase and p38 mitogen-activated protein kinase pathway in activated microglial cells. *Journal of Pharmacological Sciences*.

[31] Cheng S., Zhang C., Xu C., Wang L., Zou X., Chen G. (2014). Age-dependent neuron loss is associated with impaired adult neurogenesis in forebrain neuron-specific Dicer conditional knockout mice. *The International Journal of Biochemistry & Cell Biology*.

[32] Gerwin N., Bendele A. M., Glasson S., Carlson C. S. (2010). The OARSI histopathology initiative—recommendations for histological assessments of osteoarthritis in the rat. *Osteoarthritis and Cartilage*.

[33] Pritzker K. P. H., Gay S., Jimenez S. A. (2006). Osteoarthritis cartilage histopathology: grading and staging. *Osteoarthritis and Cartilage*.

[34] Marcu K. B., Otero M., Olivotto E., Borzi R. M., Goldring M. B. (2010). NF-*κ*B signaling: multiple angles to target OA. *Current Drug Targets*.

[35] Kim M. E., Na J. Y., Lee J. S. (2018). Anti-inflammatory effects of trans-cinnamaldehyde on lipopolysaccharide-stimulated macrophage activation via MAPKs pathway regulation. *Immunopharmacology and Immunotoxicology*.

[36] Martel-Pelletier J., Barr A. J., Cicuttini F. M. (2016). Osteoarthritis. *Nature Reviews Disease Primers*.

[37] López-Armada M. J., Caramés B., Lires-Deán M. (2006). Cytokines, tumor necrosis factor-alpha and interleukin-1beta, differentially regulate apoptosis in osteoarthritis cultured human chondrocytes. *Osteoarthritis and Cartilage*.

[38] Daheshia M., Yao J. Q. (2008). The interleukin 1*β* pathway in the pathogenesis of osteoarthritis. *The Journal of Rheumatology*.

[39] Goldring M. B., Goldring S. R. (2007). Osteoarthritis. *Journal of Cellular Physiology*.

[40] Pfander D., Heinz N., Rothe P., Carl H.-D., Swoboda B. (2004). Tenascin and aggrecan expression by articular chondrocytes is influenced by interleukin 1*β*: A possible explanation for the changes in matrix synthesis during osteoarthritis. *Annals of the Rheumatic Diseases*.

[41] Rigoglou S., Papavassiliou A. G. (2013). The NF-kappaB signalling pathway in osteoarthritis. *The International Journal of Biochemistry & Cell Biology*.

[42] Roman-Blas J. A., Jimenez S. A. (2006). NF-*κ*B as a potential therapeutic target in osteoarthritis and rheumatoid arthritis. *Osteoarthritis and Cartilage*.

[43] Raymond L., Eck S., Hays E., Tomek I., Kantor S., Vincenti M. (2007). RelA is required for IL-1*β* stimulation of Matrix Metalloproteinase-1 expression in chondrocytes. *Osteoarthritis and Cartilage*.

[44] Radons J., Bosserhoff A., Grässel S., Falk W., Schubert T. (2006). p38MAPK mediates IL-1-induced down-regulation of aggrecan gene expression in human chondrocytes. *International Journal of Molecular Medicine*.

[45] Fan Z., Söder S., Oehler S., Fundel K., Aigner T. (2007). Activation of Interleukin-1 Signaling Cascades in Normal and Osteoarthritic Articular Cartilage. *The American Journal of Pathology*.

[46] Kim B. H., Lee Y. G., Lee J., Lee J. Y., Cho J. Y. (2010). Regulatory effect of cinnamaldehyde on monocyte/macrophage-mediated inflammatory responses. *Mediators of Inflammation*.

[47] Moldovan F., Pelletier J. P., Jolicoeur F.-C., Cloutier J.-M., Martel-Pelletier J. (2000). Diacerhein and rhein reduce the ICE-induced IL-1*β* and IL-18 activation in human osteoarthritic cartilage. *Osteoarthritis and Cartilage*.

[48] Sondergaard B. C., Henriksen K., Wulf H. (2006). Relative contribution of matrix metalloprotease and cysteine protease activities to cytokine-stimulated articular cartilage degradation. *Osteoarthritis and Cartilage*.

[49] Minond D., Lauer-Fields J. L., Cudic M. (2006). The roles of substrate thermal stability and P2 and P1' subsite identity on matrix metalloproteinase triple-helical peptidase activity and collagen specificity. *The Journal of Biological Chemistry*.

[50] Garlanda C., Dinarello C. A., Mantovani A. (2013). The interleukin-1 family: back to the future. *Immunity*.

[51] Mengshol J. A., Vincenti M. P., Coon C. I., Barchowsky A., Brinckerhoff C. E. (2000). Interleukin-1 induction of collagenase 3 (matrix metalloproteinase 13) gene expression in chondrocytes requires p38, c-Jun N-terminal kinase, and nuclear factor *κ*B: differential regulation of collagenase 1 and collagenase 3. *Arthritis & Rheumatology*.

[52] Schmucker A. C., Wright J. B., Cole M. D., Brinckerhoff C. E. (2012). Distal Interleukin-1*β* (IL-1*β*) Response Element of Human Matrix Metalloproteinase-13 (MMP-13) Binds Activator Protein 1 (AP-1) Transcription Factors and Regulates Gene Expression. *The Journal of Biological Chemistry*.

[53] Meszaros E., Malemud C. J. (2012). Prospects for treating osteoarthritis: Enzyme–protein interactions regulating matrix metalloproteinase activity. *Therapeutic Advances in Chronic Disease*.

[54] Koshy P. J. T., Lundy C. J., Rowan A. D. (2002). The modulation of matrix metalloproteinase and ADAM gene expression in human chondrocytes by interleukin-1 and oncostatin M: A time-course study using real-time quantitative reverse transcription-polymerase chain reaction. *Arthritis & Rheumatology*.

[55] Zhang E., Yan X., Zhang M. (2013). Aggrecanases in the human synovial fluid at different stages of osteoarthritis. *Clinical Rheumatology*.

[56] Verma P., Dalal K. (2011). ADAMTS-4 and ADAMTS-5: key enzymes in osteoarthritis. *Journal of Cellular Biochemistry*.

[57] Guzman R. E., Evans M. G., Bove S., Morenko B., Kilgore K. (2003). Mono-iodoacetate-induced histologic changes in subchondral bone and articular cartilage of rat femorotibial joints: an animal model of osteoarthritis. *Toxicologic Pathology*.

[58] Guingamp C., Gegout-Pottie P., Philippe L., Terlain B., Netter P., Gillet P. (1997). Mono-iodoacetate-induced experimental osteoarthritis. A dose-response study of loss of mobility, morphology, and biochemistry. *Arthritis & Rheumatism*.

[59] Janusz M. J., Little C. B., King L. E. (2004). Detection of aggrecanase- and MMP-generated catabolic neoepitopes in the rat iodoacetate model of cartilage degeneration. *Osteoarthritis and Cartilage*.

[60] Gomes C., Moreira R. G., Castell-Perez E. (2011). Poly (DL-lactide-co-glycolide) (PLGA) Nanoparticles with Entrapped trans-Cinnamaldehyde and Eugenol for Antimicrobial Delivery Applications. *Journal of Food Science*.

[61] Tian W.-L., Lei L.-L., Zhang Q., Li Y. (2016). Physical Stability and Antimicrobial Activity of Encapsulated Cinnamaldehyde by Self-Emulsifying Nanoemulsion. *Journal of Food Process Engineering*.

